# Supplementary material for: Health-Related Quality of Life in Long-Term Colorectal Cancer Survivors
Source: Healthcare (Basel). 2024 Sep 25;12(19):1917. doi: 10.3390/healthcare12191917 (PMC11475455; doi:10.3390/healthcare12191917)
Supplement: Supplementary file 1 [file healthcare-12-01917-s001.zip › healthcare-3137862-supplementary.pdf]

Table S1: Description of the sociodemographic variables, tumor characteristics and treatment received at diagnosis according to followed patients and answering or not quality of life questionnaires

			Quality of Life questionnaire (N(%))			
		**Colorectal Cancer**	**Answered**	**Not participate**	**Exitus**	
		***n*= 2094**	***n*=805**	***n*=425**	***n*=864**	**p-value**
**Sex**	**Men**	1333 (63.6)	486 (60.4)	252 (59.2)	595 (68.9)	<0.001
	**Women**	761 (10.9)	319 (39.6)	173 (40.7)	269 (31.1)	
**Age at diagnosis**	**mean ± sd**	67.0 (10.9)	65.1 (10.2)	65.7 (11.1)	69.4 (10.9)	<0.001
**Age**	**≤55***	333 (15.9)	152 (18.9)	73 (17.1)	108 (12.5)	<0.001
	**56-65**	545 (26.0)	255 (31.7)	107 (25.2)	183 (21.2)	
	**66-75**	669 (32.0)	250 (31.1)	154 (36.2)	265 (30.7)	
	**76-85**	547 (26.1)	148 (18.4)	91 (21.4)	308 (35.7)	
**Area**	**Asturias**	76 (3.6)	26 (3.2)	9 (2.1)	41 (4.8)	<0.001
	**Barcelona**	684 (32.7)	212 (26.3)	177 (41.7)	295 (34.1)	
	**Cantabria**	132 (6.3)	61 (7.6)	33 (7.8)	38 (4.4)	
	**Granada**	161 (7.7)	30 (3.7)	61 (14.3)	70 (8.1)	
	**Guipúzcoa**	117 (5.6)	59 (7.3)	12 (2.8)	46 (5.3)	
	**Huelva**	71 (3.4)	34 (4.2)	2 (0.5)	35 (4.1)	
	**León**	389 (18.6)	170 (21.2)	59 (13.9)	160 (18.5)	
	**Madrid**	228 (10.9)	94 (11.7)	46 (10.8)	88 (10.2)	
	**Murcia**	34 (1.6)	7 (0.9)	8 (1.9)	19 (2.2)	
	**Navarra**	121 (5.8)	66 (8.2)	11 (2.6)	44 (5.1)	
	**Valencia**	81 (3.9)	46 (5.7)	7 (1.6)	28 (3.2)	
**Education level**	**Primary education or less**	1465 (70.0)	536 (66.6)	291 (68.3)	638 (73.8)	0.009

	**Secondary education**	417 (19.9)	182 (22.6)	94 (22.6)	141 (16.3)	
	**University**	212 (10.1)	87 (10.8)	40 (9.4)	85 (9.8)	
**Civil status**	**Single**	158 (7.6)	50 (6.2)	37 (8.7)	71 (8.2)	0.006
	**Married or Living with a partner**	1655 (79.0)	656 (81.5)	339 (79.8)	660 (76.4)	
	**Widow**	281 (13.4)	99 (12.3)	49 (11.5)	133 (15.4)	
**Smoking**	**Non-smoker and former at diagnosis***	864 (41.3)	356 (44.2)	187 (44.0)	321 (37.2)	0.006
	**Smoker at diagnosis**	1230 (58.7)	449 (55.8)	238 (56.0)	543 (62.9)	
**BMI**	**17.5-25***	631 (30.1)	254 (31.6)	126 (29.7)	251 (29.1)	0.49
	**25-29.9**	921 (44.0)	359 (44.6)	188 (44.2)	374 (43.3)	
	**≥30**	542 (25.9)	192 (23.9)	111 (26.1)	239 (27.7)	
**Family history of colon cancer**	**None**	1474 (70.4)	542 (67.3)	285 (67.1)	647 (74.9)	0.001
	**Some degree family history of colon cancer**	620 (29.6)	263 (32.7)	140 (32.9)	217 (25.1)	
**Tumor size**	**T0**	98 (4.7)	52 (6.5)	24 (5.7)	22 (2.6)	<0.001
	**T1**	125 (6.0)	61 (7.6)	27 (6.4)	37 (4.3)	
	**T2**	283 (13.5)	136 (16.9)	60 (14.1)	87 (10.1)	
	**T3**	1171 (55.9)	451 (56.0)	240 (56.5)	479 (55.4)	
	**T4**	318 (15.2)	80 (9.9)	51 (12.0)	187 (21.6)	

	**Missing**	100 (4.8)	25 (3.1)	23 (5.4)	52 (6.0)	
**Node infiltration**	**N0**	1192 (56.9)	539 (67.0)	276 (64.9)	377 (43.6)	<0.001
	**N1**	514 (24.5)	176 (21.9)	95 (22.3)	243 (28.1)	
	**N2**	286 (13.6)	64 (8.0)	41 (9.7)	180 (20.8)	
	**Missing**	103 (4.9)	26 (3.2)	13 (3.1)	64 (7.4)	
**Metastasis**	**M0**	1719 (82.1)	756 (93.9)	398 (93.7)	564 (65.3)	<0.001
	**M1**	330 (15.7)	32 (4.0)	20 (4.7)	278 (32.2)	
	**Missing**	46 (2.2)	17 (2.1)	7 (1.7)	22 (2.6)	
**Complete clinical remission***	**No**	1879 (89.7)	800 (99.4)	420 (98.8)	659 (76.3)	<0.001
	**Yes**	215 (10.3)	5 (0.6)	5 (1.2)	205 (23.7)	
	**Missing**	0	0	0	0	
**Recurrence**	**No**	1713 (81.8)	743 (92.3)	386 (90.8)	584 (67.6)	<0.001
	**Yes**	381 (18.2)	62 (7.7)	39 (9.2)	280 (32.4)	
	**Missing**	0	0	0	0	
**TNM pathological stage**	**0**	77 (3.7)	42 (5.2)	20 (4.7)	15 (1.7)	<0.001
	**I**	338 (16.1)	162 (20.1)	83 (19.5)	93 (10.8)	
	**II**	672 (32.1)	308 (38.3)	161 (37.9)	203 (23.5)	
	**III**	568 (27.1)	219 (27.2)	125 (29.4)	223 (25.8)	
	**IV**	330 (15.8)	32 (4.0)	20 (4.7)	278 (32.2)	
	**Missing**	110 (5.3)	42 (5.2)	16 (3.8)	52 (6.0)	
**Histological grade**	**Well differentiated**	522 (24.9)	249 (30.9)	82 (19.3)	191 (22.1)	<0.001
	**Moderately differentiated**	1095 (52.3)	405 (50.3)	246 (57.9)	444 (51.4)	

	**Poorly differentiated**	248 (11.8)	75 (9.3)	48 (11.3)	125 (14.5)	
	**Missing**	229 (10.9)	76 (9.4)	49 (11.5)	104 (12.0)	
**Histological type**	**Adenocarcinoma**	1879 (89.7)	737 (91.6)	374 (88.0)	768 (88.9)	0.08
	**Other**	241 (6.7)	42 (5.2)	26 (6.1)	73 (8.4)	
	**Missing**	74 (3.5)	26 (3.2)	25 (5.9)	23 (2.7)	
**Chemotherapy**	**No**	785 (37.5)	325 (40.4)	161 (37.9)	299 (34.6)	0.11
	**Yes**	1267 (60.5)	467 (58.0)	257 (60.5)	543 (62.9)	
	**Missing**	42 (2.0)	13 (1.6)	7 (1.7)	22 (2.6)	
**Radiotherapy**	**No**	1425 (68.1)	563 (69.9)	293 (68.9)	569 (65.9)	0.35
	**Yes**	524 (25.0)	194 (24.1)	104 (24.5)	226 (26.2)	
	**Missing**	145 (6.9)	48 (6.0)	28 (6.6)	69 (8.0)	
**Surgery Type**	**Radical**	1799 (85.9)	740 (91.9)	390 (91.8)	669 (77.4)	<0.001
	**Palliative**	127 (6.1)	32 (4.0)	16 (3.8)	79 (9.1)	
	**Missing**	168 (8.0)	33 (4.1)	19 (4.5)	116 (13.4)	

Table S2. Marginal means of physical component summary (PCS-12) according to sociodemographic variables, tumor characteristics and treatment received at diagnosis.

		Physical Component Summary (PCS-12)					
		Total sample (n = 805)		Men (n = 486)		Women (n = 319)	
		Mean (95% CI)	p-value (q-values)	Mean (95% CI)	p-value (q-value)	Mean (95% CI)	p-value (q- value)
**Education level**	**Primary education or less***	47.2 (46.3 - 48.1)	0.35 (0.43)	48.2 (47.1 - 49.3)	0.16 (0.33)	45.7 (44.2 - 47.1)	0.92 (0.62)
	**Secondary education**	46.7 (45.2 - 48.3)		47.7 (45.8 - 49.5)		45.8 (43.0 - 48.7)	
	**University**	48.7 (46.5 - 50.9)		50.7 (48.0 - 53.4)		44.9 (40.8 - 48.9)	
**Civil status**	**Single***	47.6 (44.7 - 50.5)	0.97 (0.63)	47.3 (43.3 - 51.4)	0.83 (0.60)	47.4 (43.0 - 51.8)	0.61 (0.53)
	**Married or Living with a partner**	47.2 (46.4 - 48.0)		48.4 (47.5 - 49.4)		45.2 (43.7 - 46.7)	
	**Widow**	47.3 (45.1 - 49.6)		47.5 (42.5 - 52.5)		46.1 (43.5 - 48.6)	
**BMI**	**17.5-25***	48.4 (47.1 - 49.7)	0.12 (0.29)	49.5 (47.8 - 51.3)	0.31 (0.40)	46.4 (44.5 - 48.4)	0.48 (0.49)
	**25-29.9**	46.7 (45.6 - 47.8)		48.0 (46.7 - 49.3)		44.7 (42.7 - 46.7)	
	**≥30**	46.8 (45.3 - 48.3)		47.8 (46.0 - 49.6)		45.8 (43.1 - 48.6)	
**Family history of colon cancer**	**None***	47.5 (46.6 - 48.4)	0.52 (0.51)	48.3 (47.2 - 49.3)	0.78 (0.58)	46.2 (44.7 - 47.7)	0.25 (0.38)
	**Some degree family history of colon cancer**	46.8 (45.6 - 48.1)		48.5 (46.9 - 50.2)		44.6 (42.6 - 46.7)	
**Tumor size**	**T0***	46.6 (39.9 - 53.2)	0.45 (0.47)	49.2 (41.5 - 56.8)	0.69 (0.55)	44.9 (31.0 - 58.8)	0.29 (0.39)
	**T1**	47.9 (44.3 - 51.6)		50.8 (46.5 - 55.1)		42.5 (35.1 - 49.8)	
	**T2**	48.8 (45.9 - 51.6)		50.5 (47.3 - 53.8)		45.1 (39.3 - 51.0)	
	**T3**	46.5 (45.1 - 47.8)		47.2 (45.5 - 48.9)		45.3 (42.8 - 47.8)	
	**T4**	48.4 (45.9 - 50.8)		47.9 (44.8 - 51.0)		49.7 (45.5 - 53.9)	
**Node infiltration**	**N0***	47.6 (45.6 - 49.6)	0.41 (0.45)	48.9 (46.5 - 51.3)	0.89 (0.62)	45.3 (41.4 - 49.2)	0.22 (0.38)
	**N1**	47.4 (43.1 - 51.7)		47.5 (42.6 - 52.5)		48.2 (39.2 - 57.2)	
	**N2**	44.8 (40.2 - 49.5)		46.3 (40.7 - 51.9)		42.7 (33.7 - 51.7)	
**Metastasis**	**M0***	47.2 (46.4 - 47.9)	0.29 (0.39)	48.3 (47.3 - 49.2)	0.61 (0.53)	45.4 (44.2 - 46.6)	0.27 (0.39)

	**M1**	48.0 (44.4 - 51.7)		48.8 (44.9 - 52.8)		48.2 (39.9 - 56.5)	
**Complete clinical remission**	**No***	47.2 (46.5 - 48.0)	0.37 (0.44)	48.3 (47.4 - 49.2)	0.31 (0.40)	-	
	**Yes**	51.7 (42.0 - 61.3)		53.3 (43.8 - 62.7)		-	
**TNM pathological stage**	**0***	45.1 (41.7 - 48.4)	0.52 (0.51)	47.0 (42.9 - 51.2)	0.92 (0.62)	42.0 (36.2 - 47.8)	0.24 (0.38)
	**I**	47.2 (45.6 - 48.8)		47.8 (45.8 - 49.8)		45.9 (43.1 - 48.7)	
	**II**	47.2 (46.0 - 48.4)		48.9 (47.4 - 50.4)		44.9 (42.9 - 46.8)	
	**III**	47.2 (45.8 - 48.6)		48.1 (46.3 - 49.9)		45.9 (43.6 - 48.2)	
	**IV**	48.0 (44.3 - 51.6)		48.8 (44.8 - 52.8)		48.1 (39.8 - 56.4)	
**Histological grade**	**Well differentiated***	46.8 (45.4 - 48.1)	0.57 (0.53)	46.9 (45.3 - 48.6)	0.13 (0.29)	46.8 (44.4 - 49.1)	0.74 (0.58)
	**Moderately differentiated**	47.5 (46.5 - 48.6)		49.2 (47.9 - 50.5)		45.0 (43.3 - 46.8)	
	**Poorly differentiated**	46.4 (44.0 - 48.8)		47.4 (44.5 - 50.3)		45.0 (40.6 - 49.3)	
**Histological type**	**Adenocarcinoma***	47.1 (46.4 - 47.9)	0.36 (0.43)	48.3 (47.4 - 49.3)	0.91 (0.62)	45.4 (44.1 - 46.6)	0.25 (0.38)
	**Other**	48.4 (45.7 – 52.3)		49.6 (45.2 - 51.8)		48.4 (43.7 - 52.4)	
**Chemotherapy**	**No***	47.4 (46.2 - 48.7)	0.93 (0.62)	48.7 (47.0 - 50.5)	0.85 (0.61)	46.0 (44.0 - 47.9)	0.86 (0.61)
	**Yes**	47.1 (46.1 - 48.2)		48.1 (46.9 - 49.3)		45.3 (43.5 - 47.1)	
**Radiotherapy**	**No***	47.6 (46.7 - 48.5)	0.38 (0.44)	48.7 (47.6 - 49.9)	0.44 (0.47)	46.0 (44.7 - 47.4)	0.38 (0.44)
	**Yes**	46.6 (45.0 - 48.1)		47.9 (46.1 - 49.6)		43.8 (41.0 - 46.6)	
	**Missing**	46.1 (43.0 - 49.2)		46.4 (42.8 - 50.1)		46.3 (40.7 - 51.8)	

Results adjusted for age at diagnosis, sex, educational level, province of recruitment, stage at diagnosis and histological grade at diagnosis analysis. * Categories used as reference in the analysis. (1) PCS-12: Physical Component Summary of SF-12, (2) MCS-12: Mental Component Summary of SF-12, (3) FCSI: a (Functional Assessment of Cancer Therapy) Colorectal Symptom Index

Table S3. Marginal means of mental component summary (MCS-12) according to sociodemographic variables, tumor characteristics and treatment received at diagnosis.

		Mental Component Summary (MCS-12)					
		Total Sample (n = 805)		Men (n = 486)		Women (n = 319)	
		Mean (95% CI)	p-value (q-value)	Mean (95% CI)	p-value (q-value)	Mean (95% CI)	p-value (q-value)
**Civil status**	**Single***	48.0 (45.1 - 50.8)	0.11 (0.29)	49.7 (45.8 - 53.6)	0.23 (0.38)	46.4 (42.0 - 50.9)	0.19 (0.34)
	**Married or Living with a partner**	50.7 (49.9 - 51.5)		51.5 (50.6 - 52.4)		49.5 (48.0 - 51.0)	
	**Widow**	49.3 (47.1 - 51.5)		55.0 (50.3 - 59.8)		47.1 (44.5 - 49.7)	
**Smoking**	**Non-smoker and former at diagnosis***	50.2 (49.1 - 51.4)	0.78 (0.58)	51.1 (49.4 - 52.8)	0.59 (0.53)	48.8 (47.3 - 50.3)	0.75 (0.58)
	**Smoker at diagnosis**	50.5 (49.5 - 51.5)		51.7 (50.7 - 52.7)		48.2 (45.5 - 50.9)	
**BMI**	**17.5-25***	50.9 (49.6 - 52.2)	0.61 (0.53)	52.1 (50.4 - 53.8)	0.64 (0.54)	49.1 (47.1 - 51.1)	0.69 (0.55)
	**25-29.9**	50.2 (49.1 - 51.3)		51.6 (50.3 - 52.8)		47.9 (45.9 - 49.9)	
	**≥30**	50.0 (48.6 - 51.5)		50.9 (49.2 - 52.6)		49.0 (46.3 - 51.8)	
**Family history of colon cancer**	**None***	50.1 (49.2 - 51.0)	0.25 (0.38)	51.2 (50.2 - 52.2)	0.28 (0.39)	48.4 (46.9 - 49.9)	0.64 (0.54)
	**Some degree family history of colon cancer**	51.0 (49.7 - 52-2)		52.3 (50.7 - 53.8)		49.0 (46.9 - 51.1)	
**Tumor size**	**T0***	53.8 (47.2 - 60.3)	0.29 (0.39)	59.1 (51.8 - 66.4)	0.17 (0.33)	41.3 (27.1 - 55.4)	0.51 (0.51)
	**T1**	52.3 (48.7 - 55.9)		52.8 (48.7 - 56.8)		52.0 (44.4 - 59.5)	
	**T2**	50.6 (47.8 - 53.3)		52.2 (49.1 - 55.3)		48.6 (42.6 - 54.5)	
	**T3**	49.7 (48.3 - 51.0)		50.5 (49.0 - 52.1)		48.5 (46.0 - 51.1)	
	**T4**	49.1 (46.7 - 51.5)		48.8 (45.9 - 51.8)		49.3 (45.0 - 53.6)	

**Node infiltration**	**N0***	49.9 (47.9 - 51.8)	0.32 (0.41)	51.2 (48.9 - 53.4)	0.26 (0.39)	47.9 (44.0 - 51.9)	0.41 (0.45)
	**N1**	51.8 (47.6 - 56.0)		53.2 (48.5 - 57.9)		49.4 (40.3 - 58.6)	
	**N2**	49.5 (44.9 - 54.1)		49.5 (44.2 - 54.9)		48.6 (39.5 - 57.8)	
**Metastasis**	**M0***	50.2 (49.5 - 50.9)	0.12 (0.29)	51.4 (50.5 - 52.2)	0.15 (0.32)	48.5 (47.3 - 49.7)	0.70 (0.55)
	**M1**	54.1 (50.5 - 57.6)		55.2 (51.4 - 59.1)		51.9 (43.4 - 60.3)	
**Complete clinical remission**	**No***	50.4 (49.7 - 51.1)	0.87 (0.61)	51.5 (50.7 - 52.4)	1.00 (0.64)	-	
	**Yes**	51.2 (41.7 - 60.6)		51.6 (42.5 - 60.6)		-	
**TNM pathological stage**	**0***	50.6 (47.3 - 53.9)	0.39 (0.44)	49.1 (45.2 - 53.1)	0.18 (0.34)	52.9 (47.0 - 58.8)	0.67 (0.55)
	**I**	49.8 (48.2 - 51.4)		51.1 (49.2 - 53.0)		47.9 (45.0 - 50.7)	
	**II**	50.6 (49.4 - 51.7)		52.2 (50.8 - 53.6)		48.1 (46.1 - 50.2)	
	**III**	50.2 (48.8 - 51.6)		51.2 (49.5 - 52.9)		48.5 (46.2 - 50.9)	
	**IV**	54.1 (50.5 - 57.6)		55.3 (51.5 - 59.1)		52.1 (43.7 - 60.6)	
**Histological grade**	**Well differentiated***	50.2 (48.8 - 51.5)	0.80 (0.59)	51.6 (50.0 - 53.1)	0.31 (0.40)	48.1 (45.7 - 50.6)	0.66 (0.55)
	**Moderately differentiated**	50.4 (49.4 - 51.4)		51.0 (49.7 - 52.2)		49.4 (47.6 - 51.2)	
	**Poorly differentiated**	49.9 (47.5 - 52.2)		51.8 (49.0 - 54.6)		47.4 (43.0 - 51.8)	
**Histological type**	**Adenocarcinoma***	50.3 (49.6 - 51.1)	0.68 (0.55)	51.5 (50.5 - 52.3)	0.38 (0.44)	48.7 (47.5 - 50.0)	0.53 (0.51)
	**Other**	51.1 (48.4 - 53.5)		53.0 (48.7 - 56.0)		47.4 (42.9 - 51.7)	
**Chemotherapy**	**No***	50.6 (49.3 - 51.8)	0.89 (0.62)	52.7 (51.0 - 54.3)	0.27 (0.39)	48.3 (46.3 - 50.2)	0.89 (0.62)
	**Yes**	50.2 (49.2 - 51.2)		50.9 (49.7 - 52.0)		48.9 (47.1 - 50.8)	
**Radiotherapy**	**No***	50.6 (49.7 - 51.4)	0.53 (0.51)	51.7 (50.6 - 52.8)	0.50 (0.51)	48.8 (47.4 - 50.2)	0.76 (0.58)
	**Yes**	49.7 (48.2 - 51.2)		50.8 (49.1 - 52.5)		47.7 (44.9 - 50.6)	
**Surgery Type**	**Radical***	50.4 (49.6 - 51.1)	0.68 (0.55)	51.6 (50.7 - 52.5)	0.17 (0.33)	48.5 (47.2 - 49.8)	0.69 (0.55)
	**Palliative**	49.2 (45.4 - 52.9)		47.6 (43.1 - 52.2)		51.5 (44.7 - 58.3)	

Results adjusted for age at diagnosis, sex, educational level, province of recruitment, stage at diagnosis and histological grade at diagnosis analysis. * Categories used as reference in the analysis

Table S4. Marginal means of functional assessment of cancer therapy colorectal symptom index (FCSI) according to sociodemographic variables, tumor characteristics and treatment received at diagnosis.

		Functional Assessment of Cancer Therapy Colorectal Sympton Index (FCSI)					
		Total Sample (n = 679)		Men (n = 398)		Women (n = 281)	
		Mean (95% CI)	p-value (q-value)	Mean (95% CI)	p-value (q-value)	Mean (95% CI)	p-value (q-value)
**Education level**	**Primary education or less***	29.1 (28.7 - 29.5)	0.55 (0.52)	29.6 (29.0 - 30.1)	0.18 (0.34)	28.4 (27.7 - 29.1)	0.61 (0.53)
	**Secondary education**	29.3 (28.6 - 30.1)		29.7 (28.8 - 30.7)		28.8 (27.4 - 30.1)	
	**University**	29.7 (28.6 - 30.9)		31.0 (29.6 - 32.4)		27.6 (25.7 - 29.6)	
**BMI**	**17.5-25***	29.2 (28.6 - 29.9)	0.98 (0.63)	30.0 (29.1 - 30.9)	0.77 (0.58)	28.1 (27.1 - 29.0)	0.56 (0.52)
	**25-29.9**	29.2 (28.6 - 29.7)		29.7 (29.1 - 30.9)		28.4 (27.5 - 29.4)	
	**≥30**	29.2 (28.5 - 30.0)		29.6 (28.7 - 30.5)		28.9 (27.7 - 30.2)	
**Family history of colon cancer**	**None***	29.2 (28.7 - 29.6)	0.80 (0.59)	29.7 (29.1 - 30.2)	0.59 (0.53)	28.4 (27.7 - 29.1)	0.85 (0.61)
	**Some degree family history of colon cancer**	29.3 (28.7 - 29.9)		30.0 (29.1 - 30.8)		28.3 (27.3 - 29.3)	
**Tumor size**	**T0***	29.7 (26.1 - 33.3)	0.24 (0.38)	30.9 (26.3 - 35.5)	0.59 (0.53)	29.7 (23.6 - 35.9)	0.43 (0.47)
	**T1**	30.3 (28.5 - 32.2)		31.0 (28.6 - 33.3)		28.6 (25.2 - 32.1)	
	**T2**	29.7 (28.3 - 31.2)		30.6 (28.8 - 32.3)		28.3 (25.6 - 31.0)	
	**T3**	28.7 (28.0 - 29.4)		29.2 (28.3 - 30.1)		27.9 (26.8 - 29.1)	
	**T4**	29.2 (28.1 - 30.4)		29.4 (27.8 - 30.9)		29.1 (27.1 - 31.0)	
**Node infiltration**	**N0***	29.1 (28.1 - 30.1)	0.55 (0.52)	29.8 (28.5 - 31.0)	0.97 (0.63)	28.0 (26.1 - 29.9)	0.38 (0.44)
	**N1**	29.8 (27.6 - 31.9)		29.9 (27.4 -32.5)		29.9 (25.6 - 34.2)	

	**N2**	28.7 (26.3 - 31.0)		29.4 (26.4 - 32.4)		27.8 (23.4 - 32.1)	
**Complete clinical remission**	**No***	29.2 (28.8 - 29.5)	0.47 (0.49)	29.8 (29.3 - 30.2)	0.44 (0.47)	-	
	**Yes**	31.0 (26.2 - 35.8)		31.7 (26.8 - 36.5)		-	
**TNM pathological stage**	**0***	29.0 (27.4 - 30.6)	0.12 (0.29)	29.5 (26.5 - 30.5)	0.33 (0.41)	30.3 (27.5 - 33.1)	0.19 (0.34)
	**I**	29.6 (28.8 - 30.4)		29.9 (28.9 - 30.9)		29.2 (27.8 - 30.5)	
	**II**	28.9 (28.4 - 29.5)		29.8 (29.0 - 30.5)		27.7 (26.8 - 28.7)	
	**III**	28.8 (28.2 - 29.5)		29.5 (28.6 - 30.4)		28.0 (26.9 -29.1)	
	**IV**	30.9 (29.0 - 32.8)		31.4 (29.3 - 33.5)		30.5 (26.2 - 34.8)	
**Histological grade**	**Well differentiated***	28.7 (28.0 - 29.4)	0.29 (0.39)	29.1 (28.2 - 29.9)	0.18 (0.34)	28.2 (27.0 - 29.3)	0.93 (0.62)
	**Moderately differentiated**	29.4 (28.9 - 29.9)		30.0 (29.4 - 30.7)		28.6 (27.8 - 29.4)	
	**Poorly differentiated**	29.0 (27.9 - 30.1)		29.5 (28.1 - 30.8)		28.5 (26.5 - 30.4)	
**Histological type**	**Adenocarcinoma***	29.2 (28.8 - 29.5)	0.59 (0.53)	29.8 (29.3 - 30.3)	0.93 (0.62)	28.3 (27.7 - 28.9)	0.45 (0.47)
	**Other**	29.6 (28.3 - 30.8)		29.7 (28.1 - 31.3)		29.2 (27.1 - 31.2)	
**Chemotherapy**	**No***	29.4 (28.7 - 30.0)	0.74 (0.58)	30.1 (29.2 - 31.0)	0.58 (0.53)	28.5 (27.6 - 29.4)	0.95 (0.62)
	**Yes**	29.1 (28.6 - 29.6)		29.6 (29.0 - 30.2)		28.3 (27.5 - 29.1)	

Results adjusted for age at diagnosis, sex, educational level, province of recruitment, stage at diagnosis and histological grade at diagnosis analysis. * Categories used as reference in the analysis. (1) PCS-12: Physical Component Summary of SF-12, (2) MCS-12: Mental Component Summary of SF-12, (3) FCSI: a (Functional Assessment of Cancer Therapy) Colorectal Symptom Index
